# Atomoxetine Does Not Improve Complex Attention in Idiopathic Parkinson's Disease Patients with Cognitive Deficits: A Meta-Analysis

**DOI:** 10.1155/2020/4853590

**Published:** 2020-02-17

**Authors:** Abhinaba Ghosh, Saibal Das, Sapan Kumar Behera, Kirubakaran Ramakrishnan, Sandhiya Selvarajan, Preeti Kandasamy, N Sreekumaran Nair

**Affiliations:** ^1^Faculty of Medicine, Biomedical Sciences (Neuroscience), Memorial University of Newfoundland, St. John's, NL, Canada; ^2^Department of Clinical Pharmacology, Jawaharlal Institute of Postgraduate Medical Education and Research (JIPMER), Puducherry, India; ^3^Department of Psychiatry, Jawaharlal Institute of Postgraduate Medical Education and Research (JIPMER), Puducherry, India; ^4^Department of Biostatistics, Jawaharlal Institute of Postgraduate Medical Education and Research (JIPMER), Puducherry, India

## Abstract

**Objectives:**

To evaluate the effects of atomoxetine on complex attention and other neurocognitive domains in idiopathic Parkinson's disease (PD).

**Methods:**

Interventional trials reporting changes in complex attention and other neurocognitive functions (Diagnostic and Statistical Manual of Mental Disorders-5) following administration of atomoxetine for at least 8 weeks in adults with idiopathic PD were included. Effect sizes (Cohen's *d*), the standardized mean difference in the scores of each cognitive domain, were compared using a random-effects model (MetaXL version 5.3).

**Results:**

Three studies were included in the final analysis. For a change in complex attention in PD with mild cognitive impairment (MCI), the estimated effect size was small and nonsignificant (0.16 (95% CI: −0.09, 0.42), *n* = 42). For changes in executive function, perceptual-motor function, language, social cognition, and learning and memory, the estimated effect sizes were small and medium, but nonsignificant. A deteriorative trend in executive function was observed after atomoxetine treatment in PD with MCI. For a change in global cognitive function in PD without MCI, the estimated effect size was large and significant.

**Conclusion:**

In idiopathic PD with MCI, atomoxetine does not improve complex attention. Also, a deteriorative trend in the executive function was noted.

## 1. Introduction

Locus coeruleus (LC) is a small pontine nucleus of about 15,000 noradrenergic neurons innervating a large number of cortical and subcortical areas in the brain, for many of which, it is the only source of norepinephrine [1]. It serves as the major noradrenergic supply to the forebrain including the prefrontal cortex and is critically important for cognitive functions [2, 3]. Loss of LC neurons has been correlated with cognitive decline in different neurodegenerative diseases [4–6]. Idiopathic Parkinson's disease (PD) is a neurodegenerative disorder, primarily characterized by bradykinesia, rigidity, and tremor [7]. It results from the degeneration of dopaminergic neurons in the substantia nigra caused by the accumulation of misfolded *α*-synuclein into Lewy neurites and Lewy bodies [7]. Interestingly, cognitive decline coarises with motor symptoms in idiopathic PD [8]. Several other clinical features throughout the different stages of idiopathic PD have been attributed to the central and peripheral norepinephrine imbalance [9]. Some of these features include autonomic and olfactory deficits, depression, and sleep disturbances along with emotional disorders [10].

Similar to other neurodegenerative diseases, loss of LC neurons has been found in idiopathic PD [11, 12]. Neuronal loss in LC correlates not only with the stage of idiopathic PD pathology [13, 14] but also with the cognitive decline [15–17]. However, as compared to the other neurodegenerative diseases, neuronal loss in LC in idiopathic PD is more extensive resulting in a widespread degeneration of the noradrenergic axons arising from it. A decline in the noradrenergic function of LC can lead to a wide array of symptoms depending upon the projection target and its function, e.g., while there is an alteration in dopamine release from both the substantia nigra and the ventral tegmental area, the clinical manifestations could be completely different. The substantia nigra-related dopamine deficiency can lead to PD but that in the ventral tegmental area would affect attention. The attention-executive function-working memory system is likely to involve the prefrontal cortex, whereas semantic memory is largely dependent on the hippocampal circuitry [18, 19]. Evidence suggests that in idiopathic PD, *α*-synuclein accumulation in LC happens earlier than in substantia nigra [11, 20, 21]. This puts LC in a critical position in the early stages of idiopathic PD, making it a potential target for treatment [22].

Norepinephrine (NE) has an important role in attention. The prefrontal cortex and parietal cortex-mediated attention systems are under strict modulation of NE. The spiking pattern of LC determines arousal state and alertness, while the depletion of NE in the forebrain leads to a lack of attention and cognitive deficit [23]. Several treatment strategies have been tried to treat cognitive impairment involving the noradrenergic system in idiopathic PD. Adrenoceptor subtype-specific agonists and antagonists [24–27], norepinephrine precursors [28–30], and both selective and nonselective norepinephrine transporter (NET) inhibitors [31–34] have been used with varied results. Atomoxetine is a selective norepinephrine reuptake inhibitor working mainly on the norepinephrine transporters at the noradrenergic axon terminals throughout the brain [35]. It is approved by the United States Food and Drug Administration for the treatment of attention-deficit hyperactivity disorder (ADHD). Atomoxetine has minimal effects on other neuromodulator receptors [36], thus qualifying as a perfect candidate for teasing out norepinephrine-specific effects in ameliorating mild cognitive impairment (MCI) in idiopathic PD. As noradrenergic axons extensively and variably innervate several areas of the brain, systemic administration of any drug manipulating it will have a mixed effect on the functions depending on the dose and area of the brain involved in that particular function [37]. This can explain why different neurocognitive functions exhibit variable effects following the administration of the same norepinephrine-modulating drug [32, 38]. The picture gets more complicated in a disease state like idiopathic PD, where more than one neuromodulatory system is affected and mutual metamodulatory effect on one system or another cannot be ruled out [9, 39].

The Diagnostic and Statistical Manual of Mental Disorders-5 (DSM-5) outlines six key neurocognitive domains, namely, executive function, perceptual-motor function, language, learning and memory, social cognition, and complex attention [19]. Neurocognitive deficit in one domain or the other should be ascribed to different brain regions and circuitries. It is to be mentioned that complex attention holds particular importance in idiopathic PD. Attention is the behavioral and cognitive process of selectively concentrating on a discrete aspect of information, whether deemed subjective or objective while ignoring other perceivable information. As per DSM-5, complex attention involves sustained attention, divided attention, selective attention, and information processing speed. Interestingly, the concentration of norepinephrine metabolite in the cerebrospinal fluid correlates well with attention and reaction time-dependent scores in idiopathic PD patients [27]. This leads to a possibility that atomoxetine may compensate for the noradrenergic deficits and improve complex attention in idiopathic PD patients.

In parallel, from a clinical point of view, it is important to understand the differential effects of noradrenergic modulation by atomoxetine on different neurocognitive domains. Multiple trials have demonstrated the beneficial effects of atomoxetine on cognitive functions, including attention, in adults with ADHD [40]. However, it is not very clear whether these beneficial effects can be extrapolated to a neurodegenerative disorder, such as idiopathic PD. Hence, this meta-analysis was conducted to evaluate the effects of atomoxetine on complex attention and other neurocognitive domains in idiopathic PD.

## 2. Materials and Methods

### 2.1. Study Design

The study protocol can be accessed in PROSPERO (CRD42018106560). Completed and published interventional trials investigating the effects of atomoxetine on cognitive functions in idiopathic PD were included. The inclusion criteria were as follows: interventional studies that included patients of age ≥18 years of either gender, diagnosed with idiopathic PD, and patients who had received atomoxetine of any dose for at least 8 weeks, and any domain of cognitive function was reported using any scale irrespective of statistical significance. The exclusion criteria were initiation or change in dose of any confounding comedication after initiating treatment with atomoxetine. The primary outcome of the study was the change in clinical score in any individual domain of cognitive function (DSM-5) [19]. Other reported neuropsychiatric assessments apart from cognitive functions were excluded from the analysis.

### 2.2. Search Strategy

MEDLINE/PubMed, IndMED, and Cochrane Library (Cochrane Database of Systematic Reviews, Cochrane Central Register of Controlled Trials (CENTRAL) and Cochrane Methodology Register) were searched until 28 November 2019. The search terms used in various combinations were “atomoxetine,” “cognition,” “cognitive therapy,” “cognitive function,” “idiopathic Parkinson's disease,” “iPD,” “Parkinson's disease,” “PD,” “neurodegenerative disease,” “mild cognitive impairment,” and “MCI.” These search terms were adapted for use with different bibliographic databases in combination with database-specific filters for studies, if available. The search strategy was used to obtain titles and abstracts of relevant studies in the English language, and they were independently screened by two authors, who subsequently retrieved abstracts, and if necessary, the full text of articles to determine suitability. Disagreement resolution was done with a third author.

### 2.3. Data Extraction and Management

The data extraction was carried out independently by two authors using a preformatted data extraction spreadsheet. No assumptions or simplifications were made during data extraction. The included studies were assessed for risk of bias by two authors independently. Categorization of the individual study reported test scales of cognitive functions into each of the six DSM-5^19^ delimited domains was performed by an experienced psychiatrist as reported in the literature [41–45]. In case of ambiguity regarding the fitness of a scale to any particular domain of cognitive function defined by DSM-5 [19], the corresponding data were excluded. The data obtained by using scales estimating the global cognitive function were analyzed separately. The direction of scoring in each scale was considered in the analysis. A random-effects model was used to ensure the robustness of the model across various population and susceptibility to outliers. Meta-analysis was performed wherever adequate data were available. Effect sizes (Cohen's *d*), the standardized mean difference in the scores of each cognitive domain, were compared in the patients using a random-effects model using MetaXL version 5.3 (© EpiGear International Pvt. Ltd.).

Attrition bias due to the amount, nature, or handling of incomplete outcome data was investigated. Attrition rate in terms of dropouts, loss to follow-up, and withdrawals were investigated. Issues of missing data and imputation methods were also critically appraised [46]. Heterogeneity was analyzed using *χ2* test on *n* − 1 degrees of freedom, with an *α* error of 5% used for statistical significance and with an *i*^2^ test [47, 48]. The *i*^2^ values of 25%, 50%, and 75% corresponded to low, medium, and high levels of heterogeneity, respectively. Effect sizes of <0.2 were considered small, 0.2–0.8 were considered medium, and >0.8 were considered large [49].

## 3. Results

**Figure 1:**
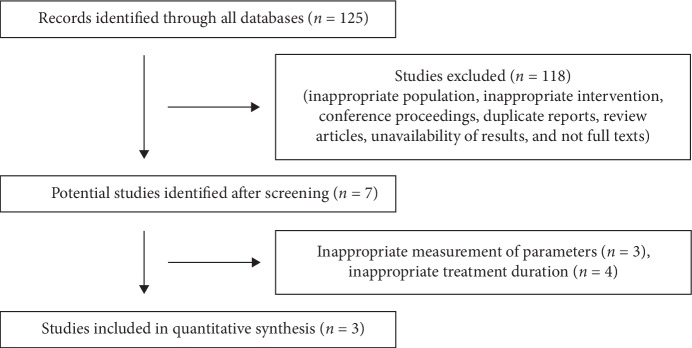
Study flowchart.

**Figure 2:**
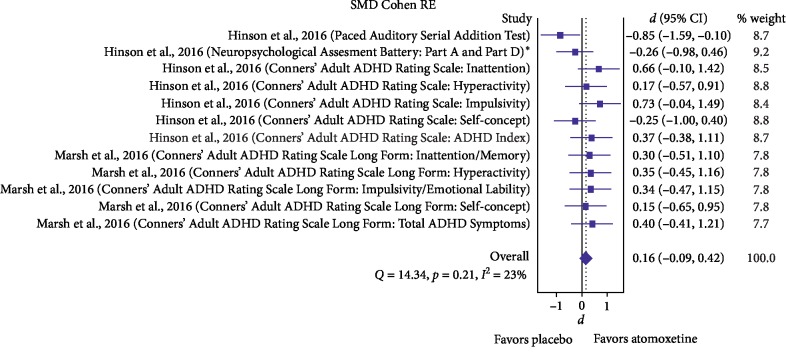
Forest plots showing the change in complex attention (DSM-5) (for Marsh et al. 2009 study, preatomoxetine data were compared to postatomoxetine data; for Hinson et al. 2016, postatomoxetine data were compared to placebo data) (ADHD: attention-deficit hyperactivity disorder). ^*∗*^The scores of Neuropsychological Assessment Battery (Part A and Part D) were combined.

A total of 125 studies were screened, and three studies [32, 38, 50] were included in the final analyses (Figure 1). Out of these three studies, two were double-blind randomized control trials and one was an open-label single-arm trial. Two of these three studies were of moderate to high quality (Supplementary [Supplementary-material supplementary-material-1]). Two studies [32, 38, 50] included PD patients with MCI. The demographic details and cognitive measurements of the included studies are enumerated in Table 1. For a change in complex attention, the estimated effect size was small and nonsignificant (0.16 (95% CI: −0.09, 0.42) (*i*^2^ = 23%, *p*=0.21), *n* = 42) (Figure 2). For a change in executive function, only one study [50] was included and the estimated effect size was medium but nonsignificant (−0.30 (95% CI: −0.62, 0.02), *n* = 30), although a trend of deterioration with atomoxetine was found (Supplementary [Supplementary-material supplementary-material-1]). For changes in perceptual-motor function (visuospatial perception), language (expressive language/confrontation naming), and social cognition (Neuropsychological Assessment Battery: Judgement), only one study [50] was included and the estimated effect sizes were small and medium, but nonsignificant (−0.06 (95% CI: −0.78, 0.65), *n* = 30; −0.12 (95% CI: −0.83, 0.60), *n* = 30; and 0.28 (95% CI: −0.44, 0.99), *n* = 30, respectively). For a change in learning and memory (Hopkins Verbal Learning Test-Revised Recognition Discrimination score), only one study [38] was included and the estimated effect size was small and nonsignificant (0.90 (95% CI: 0.06, 1.74), *n* = 12). For a change in global cognitive function (Mini-Mental State Examination (MMSE)) in PD without MCI, only one study [32] was included and the estimated effect size was large and significant (1.21 (95% CI: 0.64, 1.79), *n* = 55).

## 4. Discussion

We conducted this meta-analysis to evaluate the effects of atomoxetine on complex attention and other individual cognitive domains in idiopathic PD. We found that atomoxetine does not improve complex attention. It does not have a significant effect on other domains of cognitive function as well. Rather, a trend of deteriorating effect of atomoxetine was observed on executive function, although the results were not statistically significant. In the included studies, the patients received standard-of-care treatment, including dopaminergic drugs, and atomoxetine was used as an adjunctive agent.

A recently published systematic review [51] has efficiently summarized the effects of atomoxetine in idiopathic PD-related executive function. The authors have identified an improvement following atomoxetine administration. But such results need to be interpreted with caution. A majority (4 out of 7) of the included studies used a single dose of atomoxetine. In a chronic progressive neurodegenerative disorder, single-dose trials may lack relevance. In contrast, our study has included a minimum of 8 weeks' duration of atomoxetine administration, and further, we have evaluated the effects of atomoxetine on specific neurocognitive domains.

Although the main use and popularity of atomoxetine are generally attributed to the alleviation of ADHD, thereby rendering cognitive enhancement [52], it is understandable that the functions of atomoxetine are critically dependent on norepinephrine dynamics and noradrenergic receptor status, which can vary from disease to disease. The effects of atomoxetine on a specific disease depend on the affected brain area, its noradrenergic innervation, and the distribution of the adrenoceptors. Without these considerations, any generalization in terms of predicting the effects of atomoxetine across different diseases may go erroneous. Atomoxetine has been proven beneficial in ADHD; however, our results show that it does not improve complex attention in PD. Moreover, a trend of worsening executive function, which is also a prefrontal cortex- (PFC-) dependent function, was observed in PD patients receiving atomoxetine. This indicates that disease-specific noradrenergic pathophysiology will determine the outcome of atomoxetine treatment.

Atomoxetine prevents the reuptake of norepinephrine, thereby increasing its availability in the extracellular space for a long duration. PET studies suggest that NET deficiency occurs in PD [53] but not in ADHD [54]. Hence, in PD, atomoxetine has less amount of substrate to bind leading to a suboptimal availability of NE around the degenerating axons. In general, NE has a low affinity for *α*1 and a high affinity for *α*2 adrenoceptors. Thus, at a lower concentration of NE, *α*2 is preferentially more activated than *α*1^23^. Interestingly, the compensatory upregulation of *α*1 adrenoceptors and downregulation of *α*2 adrenoceptors have been reported in the brain in PD [55]. In general, *α*1 and *α*2 adrenoceptors have contrasting effects on PFC functioning. An increased *α*1 adrenoceptor stimulation is known to impair PFC functioning [23, 56, 57], whereas in contrast, increased *α*2 adrenoceptor functioning improves PFC function [23, 58]. Similarly, it has been reported that *α*2 antagonists can deteriorate the PFC function [59, 60]. Thus, in PD, even a suboptimal increase in the extracellular NE availability may render no effect or worsening effect due to a shifted receptor balance to increased *α*1 : *α*2 ratio. To the best of our knowledge, such a shift in the adrenoceptor ratio or noradrenergic degeneration has not been reported in ADHD. This explains why atomoxetine may remain ineffective in PD-MCI, but not in ADHD.

This may also explain why PD patients without MCI may have beneficial effects from atomoxetine. A smaller subset of PD patients does not develop MCI [61]. In the study by Weintraub et al. [32, 38, 50], the global cognitive function, as tested by MMSE, was significantly improved with a large effect size following atomoxetine treatment in PD patients without MCI. At the earliest stage of noradrenergic axon degeneration, there could still be sufficient NET left for atomoxetine to bind. Further, compensatory adrenoceptor upregulation and downregulation might not occur that early. Thus, early intervention with atomoxetine can be beneficial in PD to prevent MCI and later dementia; however, this warrants further investigations. Besides, the fact that the frontal/executive function is not covered by MMSE emphasizes the variable role of noradrenergic intervention on the different cognitive functions [62, 63]. The inability of MMSE to sensitively identify MCI in PD should be given importance. Future studies in this field might be benefitted by using an alternative test, such as the Montreal Cognitive Assessment (MoCA), instead of MMSE [64].

Our study has certain limitations. We could retrieve only three studies fulfilling the eligibility criteria, and as a result, the sample size was relatively small. The study designs also varied. Next, we could not assess the effects of atomoxetine on each domain of cognitive function due to the lack of data. Further, all the three included studies did not uniformly report data on the specific cognitive domains, and for some of the outcomes, we could include only one study. The dose and duration of atomoxetine varied. The sensitivity of the outcome measures could also be affected by the patient heterogeneity at baseline and differences in the assessment scales. [65].

## 5. Conclusion

To the best of our knowledge, this is the first meta-analysis to demonstrate that in idiopathic PD with MCI, atomoxetine does not improve complex attention, contrary to the general notion in the field. Still, long-term randomized controlled trials in a large pool of patients are necessary to further elucidate the role of atomoxetine on cognitive functions in idiopathic PD.

## Figures and Tables

**Table 1:** Characteristics of the included studies.

Author, year (country)	Type of study	Demographic details				Assessment of cognitive functions	
		Age (years) (mean ± SD)	Dose of atomoxetine (duration)	Concomitant medications	Comparator	Cognitive domain (DSM-5)	Scale and subscale used
Hinson et al. 2016 (USA)	Double-blind randomized control trial	68 ± 8	40 mg/d (weeks 1-2); 80 mg/d (weeks 3–10); no dose adjustments	Memantine, anticholinergics, MAO inhibitors, neuroleptics, and acetylcholinesterase inhibitors excluded	Placebo	Complex attention	Paced Auditory Serial Addition Test
						Complex attention	Neuropsychological Assessment Battery: Part A and Part D
						Social cognition	Neuropsychological Assessment Battery: Judgement
						Executive function	Delis–Kaplan Executive Function System—Inhibition Time
						Executive function	Delis–Kaplan Executive Function System—Inhibition-Switching Time
						Executive function	Delis–Kaplan Executive Function System—Number-Letter Switching Time
						Executive function	Delis–Kaplan Executive Function System—Proverbs
						Executive function	Wechsler Adult Intelligence Scale: Digit Span
						Language	Expressive Language/Confrontation Naming
						Perceptual-motor function	Visuospatial Perception
						Complex attention	Conners' Adult ADHD Rating Scale (Inattention)
						Complex attention	Conners' Adult ADHD Rating Scale (Hyperactivity)
						Complex attention	Conners' Adult ADHD Rating Scale (Impulsivity)
						Complex attention	Conners' Adult ADHD Rating Scale (Problems with self-concept)
						Complex attention	Conners' Adult ADHD Rating Scale (ADHD index)

Marsh et al. 2009 (USA)	Open-label single-arm trial	57.3 ± 7.2	25 mg/d (week 1), 50 mg/d (weeks 2–4), 75 mg/d (week 5), 100 mg/d (weeks 6–8); minimum 2.5 mg/d dose reductions for intolerance	Dopamine agonists, levodopa, apomorphine, COMT inhibitors, anticholinergics, selegiline, amantadine, antidepressants, atypical antipsychotics, benzodiazepine/hypnotics allowed	Pretreatment vs. posttreatment	Learning and memory	Hopkins Verbal Learning Test-Revised Recognition Discrimination score
						Complex attention	Conners' Adult ADHD Rating Scale Long Form (Inattention/Memory)
						Complex attention	Conners' Adult ADHD Rating Scales Long Form (Hyperactivity)
						Complex attention	Conners' Adult ADHD Rating Scales Long Form (Impulsivity/Emotional Lability)
						Complex attention	Conners' Adult ADHD Rating Scales Long Form (Self-concept)
						Complex attention	Conners' Adult ADHD Rating Scales Long Form (Total ADHD Symptoms)

Weintraub et al. 2010 (USA)	Double-blind randomized control trial	64.3 ± 10.5	40 mg/d (weeks 1-2), 80 mg/d (weeks 3–8); 40 mg/d allowed if indicated	Antidepressants allowed; MAO inhibitors excluded	Placebo	Global cognitive function	Mini-Mental State Examination

ADHD: attention-deficit hyperactivity disorder; COMT: catechol-O-methyl transferase; DSM-5: Diagnostic and Statistical Manual of Mental Disorders-5; MAO: monoamine oxidase; NRI: norepinephrine reuptake inhibitor.

## Data Availability

All data are included in the article.

## Supplementary Materials

Supplementary Materials Supplementary Figure 1: risk of bias summary: review authors' judgments about each risk of bias item for each included study. Supplementary Figure 2: forest plot showing the change in executive function (DSM-5).
