# Supplementary material for: Atomoxetine Does Not Improve Complex Attention in Idiopathic Parkinson's Disease Patients with Cognitive Deficits: A Meta-Analysis
Source: Parkinsons Dis. 2020 Feb 17;2020:4853590. doi: 10.1155/2020/4853590 (PMC7049416; doi:10.1155/2020/4853590)
Supplement: Supplementary Materials — Supplementary Figure 1: risk of bias summary: review authors' judgments about each risk of bias item for each included study. Supplementary Figure 2: forest plot showing the change in executive function (DSM-5). [file 4853590.f1.docx]

**Supplementary figures**

**
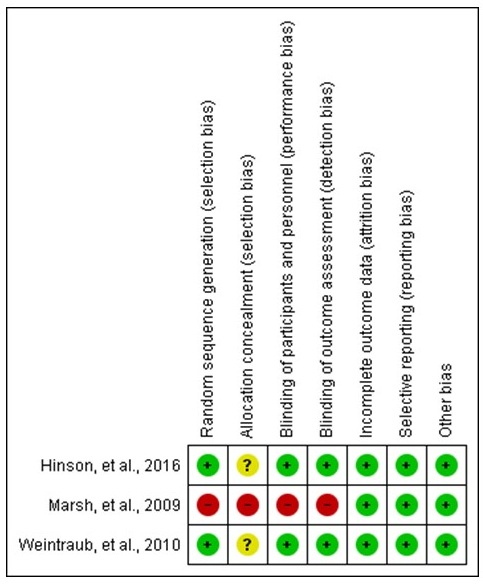
**

**Supplementary Figure 1:** Risk of bias summary: review authors' judgments about each risk of bias item for each included study.

**
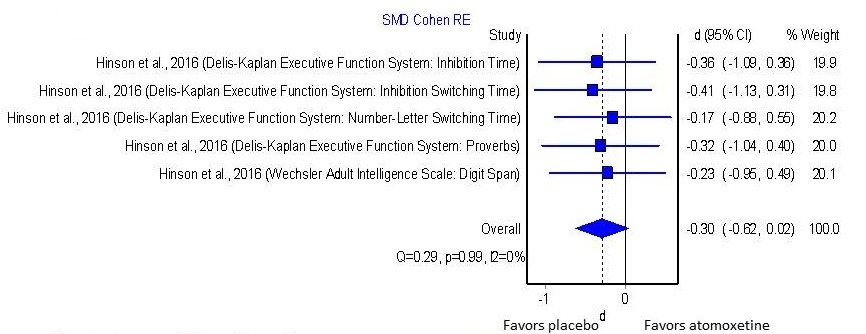
Supplementary Figure 2:** Forest plot showing the change in executive function (DSM-5).
